# The Effects of Heavy Metals and Total Petroleum Hydrocarbons on Soil Bacterial Activity and Functional Diversity in the Upper Silesia Industrial Region (Poland)

**DOI:** 10.1007/s11270-016-2966-0

**Published:** 2016-07-13

**Authors:** Beata Klimek, Anna Sitarz, Maciej Choczyński, Maria Niklińska

**Affiliations:** Institute of Environmental Sciences, Faculty of Biology and Earth Sciences, Jagiellonian University, Gronostajowa 7, 30-387 Kraków, Poland

**Keywords:** Biolog® ECO plates, Community-level physiological profiles (CLLPs), Metals, Soil microbial respiration, Total petroleum hydrocarbons (TPHs)

## Abstract

Various inorganic and organic pollutants in industrial soils may adversely affect soil microorganisms and terrestrial ecosystem functioning. The aim of the study was to explore the relationship between the microbial activity, microbial biomass, and functional diversity of soil bacteria and the metals and total petroleum hydrocarbons (TPHs) in the Upper Silesian Industrial Region (Poland). We collected soil samples in pine-dominated forest stands and analyzed them according to a range of soil physicochemical properties, including metal content (cadmium, lead, and zinc) and TPH content. Metal concentrations were normalized to their toxicity to soil microorganisms and integrated in a toxicity index (TI). Soil microbial activity measured as soil respiration rate, microbial biomass measured as substrate-induced respiration rate, and the bacterial catabolic activity (area under the curve, AUC) assessed using Biolog® ECO plates were negatively related to TPH pollution as shown in multiple regressions. The canonical correspondence analysis (CCA) showed that both TPH and TI affected the community-level physiological profiles (CLPPs) of soil bacteria and the pollutants’ effects were much stronger than the effects of other soil properties, including nutrient content.

**Electronic supplementary material:**

The online version of this article (doi:10.1007/s11270-016-2966-0) contains supplementary material, which is available to authorized users.

## Introduction

Soil microorganisms are crucial elements of terrestrial ecosystem functioning through the promotion of nutrient cycling, improvements in soil structure formation and fertility, and contributions to proper plant nutrition, controlling the energy flow to higher trophic levels in the decomposer food web (de Boer et al. 2005; Schneider et al. 2012; Massenssini et al. 2015). Soil bacteria and fungi are the main groups of organisms conducting soil organic matter (SOM) decomposition and transformations. Soil microorganisms, due to their small size, have a high surface-area-to-volume ratio and thus a large contact area with their surrounding environment (Ledin 2000). Thus, soil microorganisms are strongly susceptible to soil physicochemical properties, including the effects of various soil pollutants.

Reduced microbial activity and changes in the microbial community structure have been reported frequently in studies of metal-contaminated soils (Frostergärd et al. 1996; Chodak et al. 2013). However, in chronically polluted soils, the microorganisms can adapt to an even high concentrations of metals through various adaptive mechanisms (Piotrowska-Seget et al. 2005; Wakelin et al. 2014; Muhlbachova et al. 2015). In metal-polluted soils, the microbial response to metals may be obscured with various confounding factors, for example, soil pH or nutrient level (Azarbad et al. 2013; Chodak et al. 2013) or the simultaneous presence of other pollutants in the soil. Many of these pollutants occur in mixtures, and their effects on organisms can be antagonistic or synergistic (Baas et al. 2010). The influence of organic pollutants is especially uncertain; in contrast to metals, organic pollutants can be biotransformed by soil microorganisms into less or more toxic compounds. Moreover, microorganisms can lock them up to organic matter, reducing their bioavailability (Semple et al. 2003; Wiłkomirski et al. 2011; Sutton et al. 2013; Pacwa-Płociniczak et al. 2014). Total petroleum hydrocarbons (TPHs), defined as a mixture of aromatic and aliphatic hydrocarbons coming from crude oil products used in industry and transportation, can be both toxic for soil microorganisms as well as beneficial as a carbon source (Maliszewska-Kordybach 2003; Sutton et al. 2013). Some soil bacteria, i.e., *Pseudomonas* genus, are known to effectively degrade the hydrocarbons and are used in oil-polluted soil bioremediation (Pacwa-Płociniczak et al. 2014; de la Cueva et al. 2016).

Upper Silesia in southern Poland is one of the most contaminated areas of Europe and is often referred to as an area of ecological disaster (Pawlowski 1990). Metal ore deposits and intensive mining and smelting have resulted in high soil metal contents (Wóycicki 1913; Klimek 2012; Azarbad et al. 2013). The negative effects of metal pollution on soil microorganism activity, biomass and community functional and taxonomical indices have been shown in soils of the Upper Silesian Industrial Region (Azarbad et al. 2013; Gołębiewski et al. 2014). Among a variety of methods used to study soil microorganisms, a common method for the measuring of microbial functional diversification given as the relative degradation pattern of multiple substrates is the Biolog® ECO plate method (Preston-Mafham et al. 2002; Classen et al. 2003). Community-level physiological profiles (CLPPs) are known to reflect the environmental disturbance effects on soil bacteria well (Rutgers et al. 2016). However, a lack of metal effect on CLPP was shown in the forest soils of Upper Silesia (Azarbad et al. 2013).

The soils in Upper Silesia are polluted not only with metals but also by petroleum hydrocarbons, caused by a high level of industrialization, urbanization, and a dense road and railway network (Plaza et al. 2010). We have hypothesized that the lack of significant metal effect on bacterial CLPP in Upper Silesia indicated by Azarbad et al. (2013) might be caused by the combined influence of inorganic (metals) and organic (hydrocarbons) soil pollutants. In order to test this hypothesis, we analyzed the simultaneous effect of metal and TPH soil pollution in Upper Silesia on soil bacterial CLLP using the same method, i.e., Biolog® ECO plates.

## Materials and Methods

### Research Area and Soil Sampling

The investigations were carried out in the region of the Upper Silesia, in the two industrial areas of Olkusz and Miasteczko Śląskie. The long-term metal pollution effects on the environment in both areas are well documented (Klimek 2012; Azarbad et al. 2013). Twenty-seven soil samples were collected in forest stands located on a northern latitude from 50° 16′ to 50° 45′ and an eastern longitude from 19° 37′ to 18° 39′, including stands known to be affected by metal pollution based on earlier studies (Klimek 2012; Azarbad et al. 2013), as well as some additional, randomly distributed stands. The studied stands were chosen in order to represent a wide range of soil pollution levels. The soils were collected in forest stands dominated with pine *Pinus sylvestris* with an admixture of beech *Fagus sylvatica*, birch *Betula pendula*, larch *Larix decidua*, and oak *Quercus rubra*. Fifteen to 25 cm of upper soil layer (roughly corresponding to disturbed O soil horizon) was collected after the removal of the current year’s litter. The soils were collected during 1 day in December 2013. The samples were transported to the laboratory in plastic bags and stored at 4 °C in the dark. Then, the samples were sieved (through 5-mm mesh) to remove any stones, roots, and the green parts of plants in order to obtain homogenous samples.

### Soil Physical and Chemical Analyses

The soil dry weight (DW) was determined after drying the soil samples at 105 °C for 24 h. Water-holding capacity (WHC) was measured gravimetrically. The soil pH was measured potentiometrically in water (1:10 *w*/*v*). The C and N contents were analyzed using a CHNS analyzer (Vario EL III, Elementar Analysensysteme GmbH). The total element (Ca, K, Mg, and Na) concentrations in each soil sample were determined after wet digestion of 0.5 g of DW in 10 ml of SupraPure-concentrated HNO_3_ and HClO_4_ (7:1 *v*/*v*; Sigma-Aldrich). The concentrations of the metals (Cd, Pb, Zn) in the digests were measured using atomic absorption spectrometry (AAS) with a flame or graphite furnace nebulizer (Perkin-Elmer). The accuracy of the mineralization process was assessed using blank samples and samples of standard certified material (CRM025–050, Sandy Loam 8, RT Corp.). Because the stands are polluted with several metals (mainly Cd, Zn, and Pb), the metal pollution level in individual soil samples was expressed by a toxicity index (TI) as follows:

1 $$ \mathrm{T}\mathrm{I}={\displaystyle \sum_{i=1}^M\left(\frac{C_i}{{{\mathrm{EC}}_{50}}_i}\right)} $$

where *M* means the three studied metals, *C*_*i*_ is the concentration of *i* metal in the soil (mg kg^−1^ DW), and EC_50*i*_ is the concentration of that metal causing a 50 % reduction in dehydrogenase activity (original EC_50_s taken from Welp 1999).

TPH was measured with gas chromatography (GC-MS Clarus 600, Perkin Elmer) after extraction from 2 g of fresh soil with 5 cm^3^ of dichloromethane (DCM) in a sonic bath. The DCM phase was separated from the solids by centrifugation at 1000 RCF. Next, 2 mm^3^ of extract was transferred into the glass Pasteur pipette filled with Florisil and cleaned-up extract was collected into glass test tubes. Filtrate was evaporated to dryness at 40 °C in the stream of nitrogen; the residue was re-dissolved in 100 mm^3^ of DCM and analyzed by GC. Chromatograms were registered in the mass range of 33–500 *m*/*z*, and for quantitative determination, 57 *m*/*z* was selected. All solvents used were of GC environmental analysis grade (POCh, Poland). Anhydrous sodium sulfate (POCh) was ACS purity. The mineral oil type A and B standards (91975-F and 78473-F) and Florisil (03286-F) were purchased from Sigma-Aldrich, USA.

Each physicochemical analysis was performed in two replicates; the data were averaged and expressed based on the dry weight of the soil.

### Soil Respiration Rate and Microbial Biomass

Three sets of soil samples (equivalent of 20 g DW) were placed in plastic containers and pre-incubated for 7 days in a climate chamber at 22 ± 1 °C and 60 % WHC. The samples’ moisture was adjusted daily with deionized water. The respiration rate was measured by CO_2_ trapping (Laskowski et al. 2003). Each soil sample was placed in an airtight glass jar with a beaker of 5 ml 0.2 M NaOH. After incubation (ca. 20 h), the jars were opened and 2 ml BaCl_2_ was added to the NaOH solution; the excess sodium hydroxide was titrated using a digital Jencons burette with 0.1 M HCl (0.01-ml precision) in the presence of phenolphthalein as a color indicator. The incubation time was recorded to the nearest minute. Several empty jars (with only NaOH) were placed among the other samples as blanks. The soil respiration rate was expressed as mM CO_2_ per kg soil organic matter per 24 h. The respiration rate of each soil series was measured twice, giving six respiration measurements which were finally averaged.

Subsequently, in these same soil samples, microbial biomass was measured using substrate-induced respiration (SIR) after the addition of glucose solution (Anderson and Domsch 1978). Soil samples were amended with glucose solution corresponding to the glucose dose of 10 mg g^−1^ DW soil, which is concentration causing maximal potential respiration on glucose (data not shown). Water addition with glucose solution did not increased soil moisture above 60 % WHC. After the glucose solution addition, soils were mixed, immediately closed in jars, and incubated for 4 h at 22 °C, and soil respiration rate was measured as above. SIR was measured only once in each of soil series as effect of glucose addition to soil can be analyzed only once. Three replicate results were averaged. The microbial biomass (SIR-biomass) was calculated from the SIR according to the following equation given by Anderson and Domsch (1978): *C*_mic_ (mg g^−1^ OM) = 40.04 *y* + 0.37, where *y* was ml CO_2_ h^−1^ g^−1^ OM.

### Biolog® ECO Plate Analysis

The catabolic activity and functional diversity of the soil bacteria were analyzed using Biolog® ECO plates containing three sets of 31 carbon substrates and tetrazolium dye as the substrate utilization indicator (http://www.biolog.com). The substrates were classified into six substrate guilds, namely, amines, amino acids, carbohydrates, carboxylic acids, polymers, and miscellaneous, according to Dobranic and Zak (1999). Prior to the Biolog® analysis, the field-moist soil samples were wetted up to 60 % of their maximal water-holding capacity and pre-incubated during 1 week at 22 °C. Subsequently, the equivalent of 3 g of DW soil was shaken for 1 h in 30 ml of a 0.9 % NaCl solution (pH 7) and settled for approximately 30 min to decant the soil particles. The suspensions were diluted (10^−2^) in NaCl solution and inoculated onto Biolog® ECO plates (125 μl per well) using a multichannel pipette. All the plates were incubated in the dark at 22 °C, and substrate utilization was measured as the light absorbance at 590 nm (μQuant spectrometer; BIO-TEK Instruments). The first measurement was made immediately after inoculation, and subsequent readings were obtained at 24-h intervals for 168 h. The absorbance measurements for individual wells were corrected against the control well containing only the microbial solution. Absorbance values below 0.06 (spectrometer detection limit) were considered as 0. Each soil sample was analyzed in three replicates (one Biolog® ECO plate per sample); the data were averaged.

The general bacterial activity was calculated as the area under the curve (AUC) using the following formula:

2 $$ \mathrm{A}\mathrm{U}\mathrm{C}={\displaystyle \sum_{i=1}^N{\displaystyle \sum_{t=1}^{n-1}}\frac{\left({A}_n+{A}_{n+1}\right)}{2}\times \Big({t}_{n+1}-t{}_n}\Big) $$

where *A*_*n*_ and *A*_*n* + 1_ are the absorbance of each individual well at two consecutive measurements at times *t*_*n*_ and *t*_*n* + 1_, *n* represents the particular measurements (scorings), and *N* represents the number of substrates on the plate (31 for ECO plates; Hackett and Griffiths 1997).

The number of substrates used by the bacteria (*R*_*s*_, substrate richness) was counted for each sample. The microbial functional diversity index *H*′_bact_, derived from the Shannon-Wiener biodiversity index, which is based on the structure of substrate use

3 $$ H{\prime}_{\mathrm{bact}}=-{\displaystyle \sum_{i=1}^s{p}_s\left({ \log}_{10}{p}_s\right)}, $$

was derived from the number of substrate *s* decayed by bacteria on a set of 31 substrates and the utilization of an individual substrate *p*_*s*_ calculated as the absorbance for a given well divided by the sum of absorbance for all wells (Zak et al. 1994; Bradley et al. 2006).

Because both the density and the activity of microbial cells affect the rate of color development, the functional diversity index (*H*′_bact_) and CLPPs were compared on the same sample average well color development (AWCD), calculated as on the mean well absorbance, to compensate for differences in the initial inoculum density (AWCD = 0.15, irrespective of the incubation time; Garland 1997; Preston-Mafham et al. 2002). The absorbance values for individual wells/substrates were expressed as a proportion of the total sample absorbance on the plate, standardized to 1 for each sample.

### Statistical Analysis

Simple regression was used to show the relationship between the TI index and TPH content in the studied soils. Multiple regression analyses were used to assess the effects of soil properties on the soil respiration rate and separately, on microbial biomass, bacterial AUC, and *H*′_bact_ values. In this analysis, the respiration rate, microbial biomass, AUC, and *H*′_bact_ were used as dependent variables. The independent variables included soil C and K contents as variables representing nutrient availability for microorganisms, the TI index as a variable approximating the metal effect, and the TPH content as a variable representing the effect of petroleum products on soil microorganisms. Because the respiration rate and SIR-biomass were calculated on an OM basis, for these indices, C contents were excluded. The right-skewed data were log-transformed to fulfill the assumption of normality. The fit of the obtained models (forward factor selection) was assessed from plots of the observed versus predicted values. The percentage of total variance explained by the model was reported as the *R*^2^ value adjusted for degrees of freedom (*R*^2^_adj_).

Canonical correspondence analysis (CCA) was used to examine the correlation between the CLPP with the soil properties and pollution level on the studied stands, with backward factor selection. In this analysis, we used the C, N, and K contents and C:N ratio to represent nutrient availability and the soil wealth; the soil pH represented the soil acidity, and the TI and TPH contents represented the influence of chemical stress on CLPP.

Regression analyses were conducted using Statgraphics Centhurion XVI (StatPoint Technologies Inc., Warrenton, VA, USA) and CCA analyses using PAST 2.17c software (Natural History Museum, University of Oslo, Norway).

## Results

The collected soils were characterized by a moderate content of organic matter and biogenes and acidic to neutral soil pH (Table 1). A relatively high content of Ca and Mg resulted from the bedrock rich in alkaline minerals (Table 1). The soils were differently polluted with the three metals, and the TI index for individual soils ranged from 0.26 to as high as 50.25 (Table 1). Also, the TPH content varied strongly in the studied soils and for some soils reached the relatively high values (Table 1). A simple regression performed for the relationships between the TI index and TPH content was not significant (*p* = 0.7905).

**Table 1:** Mean values, standard deviations, and minimal and maximal values for physical, chemical, and microbial properties of studied soils (*n* = 27)

Soil property	Unit	Data set values			
		Mean	SD	Minimum	Maximum
OM	% DW	24.5	15.6	2.5	50.7
WHC	% DW	138.8	69.9	40	267
pH	–	4.83	1.06	3.70	6.70
C	% DW	16.39	10.33	1.10	33.60
N	% DW	0.56	0.30	0.06	1.11
C:N	–	26.8	7.1	14.0	36.0
Ca	% DW	0.21	0.16	0.02	0.63
K	% DW	0.11	0.10	0.03	0.48
Mg	% DW	0.05	0.04	0.01	0.16
Na	% DW	0.01	0.00	0.00	0.02
Cd	mg kg^−1^ DW	10.7	14.2	0.0	57.4
Zn	mg kg^−1^ DW	820	1376	22	5437
Pb	mg kg^−1^ DW	428	446	42	1877
TI	–	7.98	12.69	0.26	50.25
TPH	mg kg^−1^ DW	472.4	385.0	34.7	1356.5
RESP	mM CO_2_ kg^−1^ OM 24 h^−1^	28.34	16.33	11.20	94.15
SIR-biomass	mg g^−1^ OM	7.15	2.38	3.78	12.34
AUC	–	35.45	24.48	6.53	83.60
*R*_*s*_	–	27	2	23	31
*H*′_bact_	–	1.15	0.09	0.99	1.30

RESP denotes the soil respiration rate, and SIR-biomass denotes the soil microbial biomass; other shortcuts are expanded in the text

Multiple regressions yielded significant models for the soil respiration rate, soil microbial biomass, and bacterial activity AUC measured on Biolog® ECO plates (*p* values ranged from 0.0037 to 0.0261). The models explained from 15.6 % (soil respiration rate) to 28.2 % (soil microbial biomass) of the variance in these values (Table 2). The soil respiration rate, soil microbial biomass, and bacterial activity AUC were correlated to the TPH content only, and all these correlations were negative (Table 2).

**Table 2:** Relationships between values of soil respiration rate (RESP), soil microbial biomass (SIR-biomass), and bacterial AUC and selected soil physicochemical properties and pollution levels—multiple regression results (only the significant variables are presented)

Parameter	Equation parameter	*β*	*p* value	*R*_adj_^2^	*p*_mod_
RESP	Intercept	31.31	<0.0001	0.156	0.0261
	TPH	−0.01	0.0261		
SIR-biomass	Intercept	8.83	<0.0001	0.282	0.0037
	TPH	−0.003	0.0037		
AUC	Intercept	50.14	<0.0001	0.209	0.0096
	TPH	−0.03	0.0096		

The regression coefficients (*ß*) and levels of significance for the model (*p*_mod_) and for particular model parameters (*p*) are also given

The first two CCA axes calculated for the studied soils explained 53.18 % (*p* = 0.0099) and 18.97 % (*p* = 0.0297) of the variance, respectively (trace *p* = 0.0099). The first CCA axis was strongly positively related to the TPH content (0.54), a bit less to the TI (0.28) and soil pH (0.27), and negatively to the soil C:N ratio (−0.28). The largest loading to the second axis was from the TI (0.56) and TPH (−0.35).

**Fig. 1:**
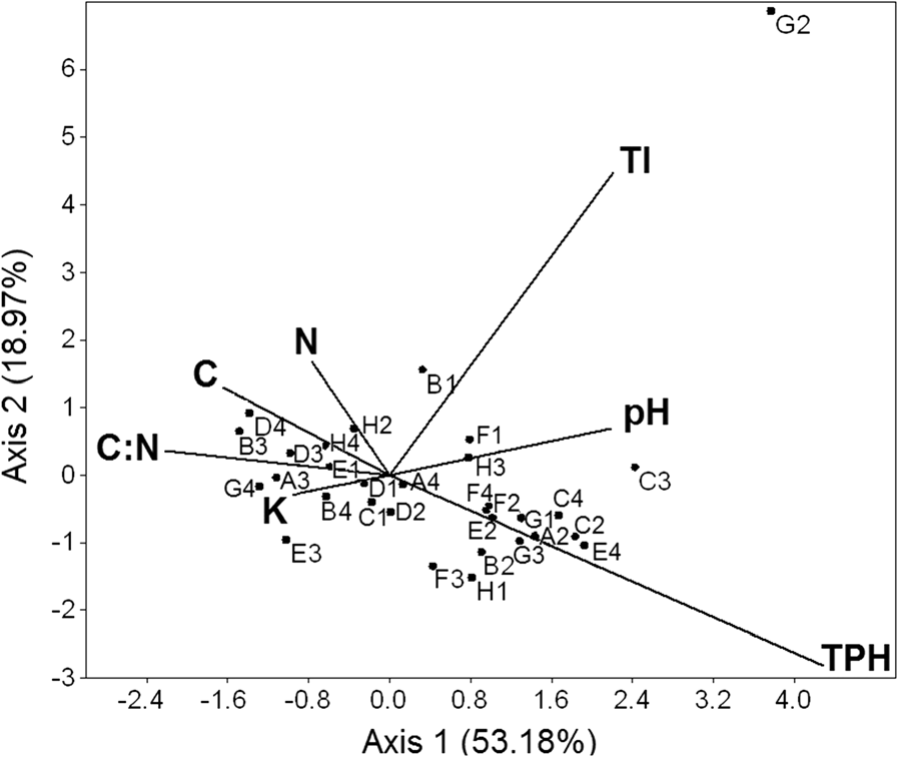
The canonical correspondence analysis (CCA) ordination plot of the bacterial substrate utilization pattern (CLPP) according to the following soil characteristics: total petroleum hydrocarbons (TPHs); metal pollution index (TI); pH; and contents of carbon (C), nitrogen (N), potassium (K); and C:N ratio. Analysis results were presented only for substrates use by soil bacteria. Substrates were denoted with *black dots* and their position on Biolog® ECO plate (*letter and number*), amines (G4-phenylethyl-amine, H4-putrescine), amino acids (A4-L-arginine, B4-L-asparagine, C4-L-phenylalanine, D4-L-serine, E4-L-threonine, F4-glycyl-L-glutamic acid), carbohydrates (G1-D-cellobiose, H1-α-D-lactose, A2-β-methyl-D-glucoside, B2-D-xylose, C2-i-erythritol, D2-D-mannitol, E2-N-acetyl-D-glucosamine), carboxylic acids (F2-D-glucosaminic acid, A3-D-galactonic acid γ-lactone, B3-D-galacturonic acid, C3-2-hydroxy benzoic acid, D3-4-hydroxy benzoic acid, E3-γ-hydroxybutyric acid, F3-itaconic acid, G3-α-ketobutyric acid, H3-D-malic acid), polymers (C1-Tween 40, D1-Tween 80, E1-α-cyclodextrin, F1-glycogen), and miscellaneous (B1-pyruvic acid methyl ester, G2-glucose-1-phosphate, H2-D,L-α-glycerol phosphate)

The CCA revealed a strong positive relationship between the use of L-threonine, i-erythritol, 2-hydroxy benzoic acid, L-phenylalanine, β-methyl-D-glucoside, α-ketobutyric acid, D-cellobiose, and soil TPH content (Fig. 1). Most of these substrates belong to easily degradable C compounds, which are carboxylic acids and carbohydrates. Strong relationships were also evident between the use of glucose-1-phosphate and the TI index (Fig. 1). The use of D-galacturonic acid, L-serine, phenylethyl-amine, and γ-hydroxybutyric acid (each representing different chemical group) was positively related to soil fertility, i.e., C, N, and K contents and C:N ratio (Fig. 1).

## Discussion

We observed a significant negative relationship between the TPH content and soil microbial activity and biomass as well as bacterial activity and functional (catabolic) soil bacterial diversity using Biolog® ECO plates. We showed that the effect of TPH exceeded the metal pollution influence, as the metals did not affect soil microbial respiration, biomass, and bacterial activity. We found that the effect of soil pH or nutrients content has also changed bacterial CLLP but did not overlay the pollutants’ effects.

The concentrations of metals measured in the studied samples were similar to those determined in earlier studies carried out in the Upper Silesian region (Klimek 2012; Azarbad et al. 2013). The metal concentrations in soils in the Upper Silesian region are as high as 57 mg Ca kg DW soil, 5437 mg Zn kg^−1^ DW soil, and 1577 mg Pb kg^−1^ DW soil.

However, we found the concentrations of TPH in the soil to be much higher than those measured by other authors (Płaza et al. 2010); nonetheless, these authors used a different method of extraction and analysis to ours. On the other hand, we found that TPH concentrations were lower than those found by Sutton et al. (2013) in a similar industrial region in Poland using the same analytical method, i.e., gas chromatography. The TPH index is often used as a common measure of environmental pollution with oil crude products, despite it being criticized because it includes the differing chemical composition of different oils, which will have different toxicities, degradability, and breakdown products (Bundy et al. 2004). Our results showed that TPH pollution in the Upper Silesian region is not correlated with metal pollution, indicating different sources and ways of dispersion of these two types of soil pollutants.

The lack of metal effect on the soil respiration rate and microbial biomass may result from high soil microbial redundancy of functions (Nannipieri et al. 2003), which allows the substitution of more sensitive to pollution microbial groups/species by those less sensitive. In long-term metal-polluted stands, soil microorganisms may acquire a tolerance to metals (Piotrowska-Seget et al. 2005; Wakelin et al. 2014; Muhlbachova et al. 2015). However, we assessed metal effect on soil microorganisms based on the total metal concentrations. It is well known that in soils with even very high total concentrations of metals, the bioavailable fraction is very small (Piotrowska-Seget et al. 2005; Muhlbachova et al. 2015), and these could result in a lack of metal effect on the soil respiration rate, microbial biomass, and some bacterial health indices measured with Biolog® ECO plates that were evaluated in our study. On the other hand, total and water-soluble metal concentrations in soils of the Upper Silesian region are usually highly correlated (Klimek and Niklińska 2007; Azarbad et al. 2013; Azarbad et al. 2015). Again, there suggest that other pollutants than metals affect negatively soil microorganisms in studied region.

Our results proved a strong effect of TPH compounds on soil microorganisms in the Upper Silesian Industrial Region. Especially, we found that TPH compounds strongly affected soil bacterial community profiles. Most of the substrates used by bacteria on Biolog® ECO plates and influenced by TPH content in soil belonged to easy degradable C compounds, which are carboxylic acids and carbohydrates. This may suggest that TPH compounds cause a shift in the bacterial community-level physiological profiles towards fast-growing bacteria orientated to a rapid use of available C compounds. This may also indicate that bacteria in TPH polluted soils are C demanding. As found earlier by Demoling et al. (2007), soil bacteria can be limited by carbon availability even in soils with a wide C:N ratio. The bacterial preference towards easily degradable C compounds on the studied soils may result from the characteristics of pine-delivered organic matter that dominated in the studied stands. It has been shown that the dominating tree species affect the soil bacterial metabolic profiles (Chodak et al. 2015; Klimek et al. 2016), and the preferential use of carboxylic acids by the microbial community was observed in pine soil (Yao et al. 2006; Chodak et al. 2015; Klimek et al. 2016).

Strong relationships were also evident between the use of glucose-1-phosphate and the soil metal pollution index. Glucose-1-phosphate is a direct product of the glycogen degradation in a reaction where glycogen phosphorylase cleaves off a molecule of glucose. The glucose-1-phosphate is thought to be a marker for intracellular catabolism (Guedon et al. 2000). This may suggest that soil bacteria in metal-polluted soils must use more C to attain the required amount of energy, in accordance with the commonly held view that stressed microorganisms divert a relatively larger amount of their available energy into maintenance of various biochemical functions (Giller et al. 1998).

The effects of TPH and metal pollution on CLPP were adverse to soil fertility and wealth index effects, including nutrient content. Therefore, we assume negative effects of TPH and metals on soil bacteria. Summing up, our results supplement the results presented by Azarbad et al. (2013), as we have shown that metal effect on bacterial CLPP in industrial region soils can be less obvious than the effect of other soil pollutant types. Further studies on this problem will have to be expanded with an analysis of the simultaneous effects of TPH and metals on the fungal part of community, as the environmental stressors can affect these soil microbial groups in different ways (Azarbad et al. 2013).

## Conclusions

We showed that TPH compounds have a negative effect on soil respiration rate, microbial biomass, and bacterial activity (AUC) measured with Biolog® ECO plates. Both TPH and metals affect the CLPPs of soil bacteria, but TPH affected CLPP more strongly than metals. Soil microorganisms in industrial regions can be subjected to various chemical pollutants, and their concentrations in soil may be not correlated.

## Electronic supplementary material

Below is the link to the electronic supplementary material.

ESM 1 (DOCX 31 kb)

## References

[CR1] Anderson JPE, Domsch KH (1978). A physiological method for the quantitative measurement of microbial biomass in soils. Soil Biology and Biochemistry.

[CR2] Azarbad H, Niklińska M, van Gestel CAM, van Straalen NM, Röhling WFM, Laskowski R (2013). Microbial community structure and functioning along metal pollution gradients. Environmental Toxicology and Chemistry.

[CR3] Azarbad H, Niklińska M, Nikiel K, van Straalen NM, Röling WFM (2015). Functional and compositional responses in soil microbial communities along two metal pollution gradients: does the level of historical pollution affect resistance against secondary stress?. Biology and Fertility of Soils.

[CR4] Baas J, Stefanowicz AM, Klimek B, Laskowski R, Kooijman SALM (2010). Model-based experimental design for assessing effects of mixtures of chemicals. Environmental Pollution.

[CR5] Bradley RL, Shipley B, Beaulieu C (2006). Refining numerical approaches for analyzing soil microbial community catabolic profiles based on carbon source utilization patterns. Soil Biology and Biochemistry.

[CR6] Bundy JG, Paton GI, Campbell CD (2004). Combined microbial community level and single species biosensor responses to monitor recovery of oil polluted soil. Soil Biology and Biochemistry.

[CR7] Chodak M, Gołębiewski M, Morawska-Płoskonka J, Kuduk K, Niklińska M (2013). Diversity of microorganisms from forest soils differently polluted with heavy metals. Applied Soil Ecology.

[CR8] Chodak M, Pietrzykowski M, Sroka K (2015). Physiological profiles of microbial communities in mine soils afforested with different tree species. Ecological Engineering.

[CR9] Classen AT, Boyle SI, Haskins KE, Overby ST, Hart SC (2003). Community-level physiological profiles of bacteria and fungi: plate type and incubation temperature influences contrasting soils. FEMS Microbiology Ecology.

[CR10] de Boer W, Folman LB, Summerbell RC, Boddy L (2005). Living in a fungal world: impact of fungi on soil bacterial niche development. FEMS Microbiology Reviews.

[CR11] de la Cueva SC, Rodríguez CH, Soto Cruz NO, Rojas Contreras JA, López Miranda J (2016). Changes in bacterial populations during bioremediation of soil contaminated with petroleum hydrocarbons. Water, Air, and Soil Pollution.

[CR12] Demoling F, Figueroa D, Bååth E (2007). Comparison of factors limiting bacterial growth in different soils. Soil Biology and Biochemistry.

[CR13] Dobranic JK, Zak JC (1999). A microtiter plate procedure for evaluating fungal functional diversity. Mycologia.

[CR14] Frostergärd Å, Tunlid A, Bååth E (1996). Changes in microbial community structure during long-term incubation in two soils experimentally contaminated with metals. Soil Biology and Biochemistry.

[CR15] Garland JL (1997). Analysis and interpretation of community-level physiological profiles in microbial ecology. FEMS Microbiology Ecology.

[CR16] Giller KE, Witter E, McGrath SP (1998). Toxicity of heavy metals to microorganisms and microbial processes in agricultural soils: a review. Soil Biology and Biochemistry.

[CR17] Gołębiewski M, Deja-Sikora E, Cichosz M, Tretyn A, Wróbel B (2014). 16S rDNA pyrosequencing analysis of bacterial community in heavy metals polluted soils. Microbial Ecology.

[CR18] Guedon E, Desvaux M, Petitdemange H (2000). Kinetic analysis of *Clostridium cellulolyticum* carbohydrate metabolism: importance of glucose 1-phosphate and glucose 6-phosphate branch points for distribution of carbon fluxes inside and outside cells as revealed by steady-state continuous culture. Journal of Bacteriology.

[CR19] Hackett CA, Griffiths BS (1997). Statistical analysis of the time-course of Biolog substrate utilization. Journal of Microbial Methods.

[CR20] Klimek B, Niklińska M (2007). Zinc and copper toxicity to soil bacteria and fungi from zinc polluted and unpolluted soils: a comparative study with different types of Biolog plates. Bulletin of Environmental Contamination and Ecology.

[CR21] Klimek B (2012). Effect of long-term zinc pollution on soil microbial community resistance to repeated contamination. Bulletin of Environmental Contamination and Toxicology.

[CR22] Klimek B, Chodak M, Jaźwa M, Solak A, Tarasek A, Niklińska M (2016). The relationship between soil bacteria substrate utilisation patterns and the vegetation structure in temperate forests. European Journal of Forest Research.

[CR23] Laskowski R, Niklińska M, Nycz-Wasilec P, Wójtowicz M, Weiner J (2003). Variance components of the respiration rate and chemical characteristics of soil organic layers in Niepołomice Forest, Poland. Biogeochemistry.

[CR24] Ledin M (2000). Accumulation of metals by microorganisms—processes and importance for soil systems. Earth Sciences Reviews.

[CR25] Maliszewska-Kordybach B, Smreczak B (2003). Habitat function of agricultural soils as affected by heavy metals and polycyclic aromatic hydrocarbons contamination. Environment International.

[CR26] Massenssini AM, Bonduki VHA, Melo CAD, Tótola MR, Ferreira FA, Costa MD (2015). Relative importance of soil physico-chemical characteristics and plant species identity to the determination of soil microbial community structure. Applied Soil Ecology.

[CR27] Muhlbachova G, Sagova-Mareckova M, Omelka M, Szakova J, Tlustos P (2015). The influence of soil organic carbon on interactions between microbial parameters and metal concentrations at a long-term contaminated site. The Science of the Total Environment.

[CR28] Nannipieri P, Ascher J, Ceccherini MT, Landi L, Pietramellara G, Renella G (2003). Microbial diversity and soil functions. European Journal of Soil Science.

[CR29] Pacwa-Płociniczak M, Płaza GA, Poliwoda A, Piotrowska-Seget Z (2014). Characterization of hydrocarbon-degrading and biosurfactant-producing *Pseudomonas* sp. P-1 strain as a potential tool for bioremediation of petroleum-contaminated soil. Environmental Science and Pollution Research.

[CR30] Pawlowski L (1990). Chemical threat to the environment in Poland. The Science of the Total Environment.

[CR31] Piotrowska-Seget Z, Cycoń M, Kozdrój J (2005). Metal-tolerant bacteria occurring in heavily polluted soil and mine spoil. Applied Soil Ecology.

[CR32] Płaza G, Nałęcz-Jawecki G, Pinyakong O, Illmer P, Margesin R (2010). Ecotoxicological and microbiological characterization of soils from heavy-metal- and hydrocarbon contaminated sites. Environmental Monitoring Assessment.

[CR33] Preston-Mafham J, Boddy L, Randerson PF (2002). Analysis of microbial community functional diversity using sole-carbon-source utilisation profiles—a critique. FEMS Microbiology Ecology.

[CR34] Rutgers M, Wouterse M, Drost SM, Breure AM, Mulder C, Stone D, Creamer RE, Winding A, Bloemt J (2016). Monitoring soil bacteria with community-level physiological profiles using Biolog^™^ ECO-plates in the Netherlands and Europe. Applied Soil Ecology.

[CR35] Schneider T, Keiblinger KM, Schmid E, Sterflinger-Gleixner K, Ellersdorfer G, Roschitzki B, Richter A, Eberl L, Zechmeister-Boltenstern S, Riedel K (2012). Who is who in litter decomposition? Metaproteomics reveals major microbial players and their biogeochemical functions. The ISME Journal.

[CR36] Semple KT, Morris AWJ, Paton GI (2003). Bioavailability of hydrophobic organic contaminants in soils: fundamental concepts and techniques for analysis. European Journal of Soil Science.

[CR37] Sutton NB, Maphosa F, Morillo JA, Abu Al-Soud W, Langenhoff AAM, Grotenhuis T, Rijnaarts HHM, Smidt H (2013). Impact of long-term diesel contamination on soil microbial community structure. Applied and Environmental Microbiology.

[CR38] Wakelin S, Gerard E, Black A, Hamonts K, Condron L, Yuan T, van Nostrand J, Zhou J, O’Callaghan M (2014). Mechanisms of pollution induced community tolerance in a soil microbial community exposed to Cu. Environmental Pollution.

[CR39] Welp G (1999). Inhibitory effects of the total and water-soluble concentrations of nine different metals on the dehydrogenase activity of a loess soil. Biology and Fertility of Soils.

[CR40] Wiłkomirski B, Sudnik-Wójcikowska B, Galera H, Wierzbicka M, Malawska M (2011). Railway transportation as a serious source of organic and inorganic pollution. Water, Air, and Soil Pollution.

[CR41] Wóycicki Z (1913). Vegetation in the Kingdom of Poland IV. The calamine flora of Boleslaw and Olkusz.

[CR42] Yao H, Bowman D, Shi W (2006). Soil microbial community structure and diversity in a turfgrass chronosequence: land-use change versus turfgrass management. Applied Soil Ecology.

[CR43] Zak JC, Willig MR, Moorhead DL, Wildmand HG (1994). Functional diversity of microbial communities: a quantitative approach. Soil Biology and Biochemistry.

