# Supplementary material for: The Effects of Heavy Metals and Total Petroleum Hydrocarbons on Soil Bacterial Activity and Functional Diversity in the Upper Silesia Industrial Region (Poland)
Source: Water Air Soil Pollut. 2016 Jul 13;227:265. doi: 10.1007/s11270-016-2966-0 (PMC4943982; doi:10.1007/s11270-016-2966-0)
Supplement: Supplementary file 1 — (DOCX 31 kb) [file 11270_2016_2966_MOESM1_ESM.docx]

Electronic Suplementary Material 1

Article title: “The Effects of Heavy Metals and Total Petroleum Hydrocarbons on Soil Bacteria Activity and Functional Diversity in the Upper Silesia Industrial Region (Poland)”

Journal: “Water, Air and Soil Pollution”

Authors: Beata Klimek*, Anna Sitarz, Maciej Choczyński, Maria Niklińska

*- corresponding author, e-mail: [beata.klimek@uj.edu.pl](mailto:beata.klimek@uj.edu.pl)

| Soil property | Unit | Soil sample number | | | | | | | | | | | | | | | | | | | | | | | | | | |
| --- | --- | --- | --- | --- | --- | --- | --- | --- | --- | --- | --- | --- | --- | --- | --- | --- | --- | --- | --- | --- | --- | --- | --- | --- | --- | --- | --- | --- |
|  |  | 1 | 2 | 3 | 4 | 5 | 6 | 7 | 8 | 9 | 10 | 11 | 12 | 13 | 14 | 15 | 16 | 17 | 18 | 19 | 20 | 21 | 22 | 23 | 24 | 26 | 26 | 27 |
| OM | % DW | 4 | 4 | 3 | 10 | 18 | 6 | 12 | 16 | 24 | 32 | 22 | 23 | 22 | 26 | 49 | 7 | 45 | 21 | 48 | 49 | 20 | 33 | 39 | 15 | 19 | 51 | 46 |
| WHC | % DW | 48 | 45 | 40 | 79 | 125 | 48 | 100 | 129 | 161 | 195 | 118 | 158 | 104 | 149 | 217 | 46 | 267 | 128 | 224 | 246 | 105 | 177 | 158 | 79 | 108 | 257 | 237 |
| pH | - | 6.67 | 5.66 | 5.40 | 4.05 | 4.68 | 4.45 | 4.08 | 4.11 | 6.52 | 3.71 | 3.80 | 4.45 | 6.43 | 6.14 | 6.22 | 3.89 | 4.07 | 4.08 | 3.96 | 3.70 | 5.91 | 5.94 | 6.11 | 3.73 | 3.91 | 4.94 | 3.82 |
| C | % DW | 2.39 | 18.56 | 19.89 | 32.75 | 2.87 | 18.67 | 21.71 | 33.6 | 3.6 | 28.37 | 22.72 | 12.05 | 5.47 | 1.1 | 2.05 | 14.43 | 6.32 | 28.49 | 23.65 | 28.77 | 11.82 | 10.82 | 6.02 | 17.17 | 31.04 | 16.99 | 21.21 |
| N | % DW | 0.16 | 0.72 | 0.78 | 0.96 | 0.15 | 0.63 | 0.62 | 1.11 | 0.22 | 0.81 | 0.68 | 0.43 | 0.22 | 0.06 | 0.14 | 0.40 | 0.30 | 0.99 | 0.79 | 0.79 | 0.53 | 0.52 | 0.37 | 0.49 | 1.00 | 0.69 | 0.63 |
| C:N | - | 15 | 26 | 26 | 34 | 20 | 29 | 35 | 30 | 16 | 35 | 34 | 28 | 25 | 20 | 14 | 36 | 21 | 29 | 30 | 36 | 22 | 21 | 16 | 35 | 31 | 25 | 34 |
| Ca | % DW | 0.14 | 0.43 | 0.33 | 0.07 | 0.03 | 0.14 | 0.02 | 0.24 | 0.34 | 0.17 | 0.08 | 0.13 | 0.44 | 0.03 | 0.18 | 0.13 | 0.05 | 0.15 | 0.18 | 0.21 | 0.63 | 0.55 | 0.42 | 0.10 | 0.29 | 0.06 | 0.21 |
| K | % DW | 0.05 | 0.12 | 0.16 | 0.03 | 0.18 | 0.10 | 0.05 | 0.12 | 0.06 | 0.08 | 0.08 | 0.06 | 0.08 | 0.03 | 0.11 | 0.07 | 0.07 | 0.07 | 0.48 | 0.07 | 0.08 | 0.15 | 0.08 | 0.07 | 0.38 | 0.20 | 0.06 |
| Mg | % DW | 0.03 | 0.06 | 0.06 | 0.01 | 0.03 | 0.05 | 0.01 | 0.05 | 0.10 | 0.03 | 0.02 | 0.03 | 0.11 | 0.01 | 0.04 | 0.02 | 0.02 | 0.06 | 0.04 | 0.03 | 0.16 | 0.15 | 0.08 | 0.02 | 0.06 | 0.01 | 0.02 |
| Na | % DW | 0.01 | 0.01 | 0.02 | 0.00 | 0.00 | 0.01 | 0.00 | 0.01 | 0.01 | 0.01 | 0.01 | 0.00 | 0.01 | 0.00 | 0.01 | 0.00 | 0.00 | 0.01 | 0.01 | 0.00 | 0.01 | 0.01 | 0.01 | 0.01 | 0.01 | 0.00 | 0.00 |
| Cd | mg kg^-1^ DW | 22.5 | 17.5 | 11.4 | 1.6 | 0.4 | 7.6 | 0.0 | 9.7 | 24.4 | 2.8 | 1.5 | 4.3 | 48.5 | 3.4 | 11.6 | 1.6 | 0.1 | 7.6 | 2.7 | 4.2 | 57.4 | 20.3 | 14.3 | 0.6 | 9.1 | 1.1 | 4.1 |
| Zn | mg kg^-1^ DW | 3277 | 1028 | 432 | 50 | 22 | 303 | 33 | 489 | 1763 | 104 | 76 | 182 | 5437 | 280 | 879 | 62 | 48 | 401 | 93 | 178 | 4338 | 1015 | 1161 | 65 | 270 | 59 | 105 |
| Pb | mg kg^-1^ DW | 449 | 630 | 579 | 152 | 42 | 612 | 135 | 921 | 752 | 270 | 184 | 178 | 1588 | 204 | 361 | 125 | 117 | 736 | 226 | 254 | 1877 | 705 | 775 | 178 | 493 | 65 | 150 |
| TI | - | 29.45 | 10.10 | 4.78 | 0.69 | 0.27 | 3.67 | 0.51 | 5.78 | 16.77 | 1.38 | 0.97 | 1.94 | 50.28 | 2.84 | 8.43 | 0.77 | 0.67 | 4.74 | 1.27 | 2.10 | 41.31 | 10.21 | 11.51 | 0.88 | 3.25 | 0.73 | 1.25 |
| TPH | mg kg^-1^ DW | 224 | 35 | 37 | 51 | 87 | 56 | 126 | 85 | 170 | 355 | 223 | 323 | 332 | 616 | 1154 | 282 | 807 | 489 | 975 | 1356 | 807 | 800 | 706 | 310 | 547 | 1112 | 690 |
| RESP | mM CO_2_ kg^-1^ OM 24 h^-1^ | 38.75 | 58.86 | 94.15 | 29.35 | 31.93 | 29.95 | 16.17 | 19.72 | 23.13 | 30.44 | 19.25 | 32.27 | 26.23 | 26.49 | 15.99 | 38.45 | 22.59 | 19.26 | 27.45 | 19.78 | 26.61 | 14.76 | 11.20 | 18.46 | 33.71 | 20.33 | 19.93 |
| SIR-biomass | mg g^-1^ OM | 9.44 | 7.44 | 4.72 | 6.79 | 10.92 |  | 8.90 | 9.92 | 6.07 | 6.34 | 7.93 | 7.51 | 8.64 | 7.04 | 3.76 |  | 4.68 | 7.74 | 5.06 | 4.58 | 9.55 | 5.29 | 4.92 | 12.34 | 10.39 | 4.33 | 4.57 |
| AUC | - | 50.20 | 9.75 | 22.41 | 64.10 | 60.91 | 23.87 | 83.60 | 66.70 | 82.05 | 63.72 | 56.31 | 46.23 | 18.50 | 17.15 | 12.31 | 41.80 | 12.09 | 13.52 | 35.37 | 16.95 | 10.10 | 8.62 | 6.53 | 7.29 | 56.63 | 27.86 | 42.49 |
| R_s_ | - | 23 | 29 | 24 | 26 | 25 | 28 | 26 | 26 | 26 | 23 | 29 | 30 | 27 | 24 | 25 | 23 | 31 | 27 | 28 | 27 | 26 | 29 | 26 | 29 | 30 | 26 | 27 |
| H’_bact_ | - | 1.14 | 1.24 | 0.99 | 1.17 | 1.15 | 1.10 | 1.20 | 1.12 | 1.22 | 1.08 | 1.18 | 1.15 | 1.02 | 1.03 | 1.24 | 1.01 | 1.29 | 1.04 | 1.22 | 1.08 | 1.18 | 1.30 | 1.27 | 1.24 | 1.20 | 1.07 | 1.10 |
